# Contending with Spiritual Reductionism: Demons, Shame, and Dividualising Experiences Among Evangelical Christians with Mental Distress

**DOI:** 10.1007/s10943-021-01268-9

**Published:** 2021-05-15

**Authors:** Christopher E. M. Lloyd

**Affiliations:** Human Sciences Research Centre, University of Derby, Kedleston Road, Derby, DE22 1GB UK

**Keywords:** Christianity, Demons, Evangelicalism, Mental health, Stigma, Shame, Qualitative, Interpretative phenomenological analysis (IPA)

## Abstract

The belief that mental distress is caused by demons, sin, or generational curses is commonplace among many evangelical Christian communities. These beliefs may have positive or negative effects for individuals and groups. Phenomenological descriptions of these experiences and the subjective meanings associated with them, however, remain somewhat neglected in the literature. The current study employed semi-structured interviews with eight evangelical Christians in order to idiographically explore their experiences of mental distress in relation to their faith and wider communities. Through an interpretative phenomenological analysis, two superordinate themes were constructed: negative spiritualisation and negotiating the dialectic between faith and the lived experience of mental distress. Participants variously experienced a climate of negative spiritualisation, whereby their mental distress was demonised and dismissed, and they were further discouraged from seeking help in secular institutions and environments. Participants often considered such dismissals of their mental distress as unhelpful and stigmatising and experienced heightened feelings of shame and suffering as a result. Such discouragement also contributed to the process of othering and relational disconnection. Alongside a rejection of church teachings, which exclusively spiritualised psychological distress, participants negotiated a nuanced personal synthesis of faith, theology, and distress, which assumed a localised and idiographic significance. This synthesis included advocating for the uptake of aetiological accounts, which contextualised mental distress in terms of the whole person and resisted de-politicised, dichotomised, and individualistic narratives. Results are discussed in relation to a broad range of literature in the field, while further research suggestions are provided.

## Early Pastoral Psychology

Long before modern psychotherapy was developed, the “curing of souls” had been central to the church’s mission (Bergin, 1991). As naturalistic ontologies and epistemologies have permeated the broader culture, theological studies have increasingly been rejected as legitimate sources of knowledge for human nature and suffering, with preference given to empirical studies concerning the senses, mind, memory, and behaviour (Yarhouse et al., 2016). As Lloyd and Waller (2020) suggest, this has resulted in a historically hostile relationship between Christianity and psychotherapeutic care, often characterised by reciprocal distrust (Harley, 2007; Kay and Parry, 2011; Poole and Cook, 2011).^1^ This conflictual dynamic has culminated in a false split between spiritual intervention and secular care (Webb et al., 2008).

### Links Between Religion and Well-being

This divide is especially alarming considering a substantial body of literature confirming the positive effects of religious belief and practice on psychological well-being (Koenig, 2012; Pargament, 1997; Yangarber-Hick, 2004). Indeed, psychological well-being is closely interconnected with an individual's religious belief system and may be an important coping strategy for mental distress, providing meaning, social support, and hope (Bonelli and Koenig, 2013; Pargament et al., 2013).

Pargament et al. (1998, 2011) distinguishes between positive and negative religious coping by defining positive religious coping strategies such as interpreting a difficult experience as potentially transformative, whereas negative coping strategies include reinterpreting experiences as a vengeance by God. Specifically, positive coping may include treating God as a personal guide, pursuing and valuing God's care and love, and establishing “a secure relationship with a transcendent force, a sense of spiritual connectedness with others, and a benevolent world view” (Pargament et al., 2011, p. 51). Accordingly, positive religious coping strategies tend to be adaptive for individuals under physical or psychological pressure. By contrast, individuals who adopt negative religious coping strategies may passively rely on God to remedy difficulties and strive to cope without trusting God's help. Such strategies may represent “underlying spiritual tensions and struggles within oneself, with others, and with the divine” (Pargament et al., 2011, p. 51).

The link between religion and well-being, however, is multifaceted and often assumes a localised, subjective meaning (Abu-Raiya et al., 2016). For individuals and groups with mental distress in spiritual communities, an important consideration influencing whether religion bolsters well-being is the particular religious beliefs assumed about mental distress, comprising aetiological factors and preferred treatment options (Laythe et al., 2002; Leavey, 2010; Leavey et al., 2016).

### Christianity, Theological Belief, and Mental Distress

Christian communities commonly view emotional and mental health as reflective of the soul and spirit’s internal workings (Cook and Hamley, 2020; Scrutton, 2020; Webb, 2017). This is especially true for evangelical Christianity,^2^ which is defined as a transdenominational movement, emphasising personal conversion, the absolute authority of the Bible, a dual focus on the doctrinal implication of Jesus’s death and resurrection, and the importance of Evangelism for all individuals (Bebbington, 2003). In addition to an emphasis on Jesus’s death and his resurrection, adherence to a pneumatological soteriology (salvation as the result of both Christ and Holy Spirt) is central (Ngong, 2010), whereby the Holy Spirit (one of the three divine persons within the Holy Trinity) is understood to facilitate discernment of the spiritual kingdom, speaking in tongues (glossolalia), and healing (Tidball, 1994).

Within such communities, spiritualised aetiologies for mental distress may take on heightened prominence (Lloyd and Hutchinson, in press; Weaver, 2014). For instance, in evangelical groups (including Pentecostalism), the term “spiritual warfare” is routinely employed to denote an ongoing battle between God and Satanic forces that seek to damage and destroy the lives of humans (Nie and Olson, 2016). Members or leaders of Christian communities may embolden individuals to pursue spiritual intervention where this is doctrinally advocated (e.g., prayer, fasting, healing, or deliverance), or to avoid medical or secular treatments (e.g., medication, talking therapy) and focus instead on pursuing remedies solely directed towards spiritual health (Lloyd and Hutchinson, in press; Malony, 1998; Stanford, 2007). Furthermore, experiences of mental distress may be implicitly or explicitly equated to demonic infiltration or possession, generational curses, a sinful lifestyle, or lacking faith (Dobbins, 2000; Hammond and Hammond, 1973; Hunt, 1998; for a review, see Mercer, 2013). Indeed, quantitative research undertaken in the U.S. suggests that belief in demons can lead to diminished mental health. Importantly, low mental health in itself was not found to predict greater belief in demons, which suggests a potential negative influence for subscribing to demonic aetiologies (Nie and Olson, 2016).

The potential pathways for how belief in the demonic impacts upon wellbeing are debateable, but the literature purports that understandings of mental distress as exclusively demonic in aetiology or as the consequence of unrepented sin (Scrutton, 2015, 2020) may stigmatise vulnerable populations (Weaver, 2014). Scrutton (2020) argues that such accounts are often damaging, as they potentially de-politicise the social, relational, and political context of mental distress and overemphasise individual responsibility. Indeed, overreliance on sin or demonic accounts of mental distress may contribute to what has been termed a “dividualising” process, through which an individual’s identity becomes isolated from their life context, meaning and experience (Colwell, 1996).

While the deterministic belief that psychological suffering is solely indicative of the demonic has recently been subject to fierce appraisal and critique from the theological academy (e.g., Cook and Hamley, 2020; Scrutton, 2020; Webb, 2017), we currently lack phenomenological descriptions from individuals with lived experiences of mental distress to explore such claims.

### Understandings of Evangelical Faith and Mental Distress

Previous research has adopted a mostly nomothetic perspective to survey the experiences of those with mental distress in the Evangelical community. In a survey by Stanford (2007), over 30% of 293 participants reported a negative church interaction in the context of their mental distress. Negative interactions included being rejected by the church, broader teachings that mental distress was exclusively associated with the work of demons, and that mental distress was the consequence of personal sin. In a comparable survey of 85 Christians, over 40% described having their psychiatric diagnoses dismissed by the church (Stanford and McAlister, 2008). Similarly, Hartog and Gow (2005) surveyed 126 Protestant Christians whereby 38% endorsed a demonic aetiology for major depression, while 37% endorsed a demonic aetiology for schizophrenia. Both of these findings are broadly analogous to a recent large-scale quantitative survey of 446 respondents by Lloyd and Waller (2020) in the UK. In their study, 31% of respondents stated experiencing church teaching, which exclusively spiritualised their mental distress by equating it as caused by demons, sin, or generational curses. In articulating their own aetiological conceptualisations of mental distress, furthermore, 73% endorsed non-spiritual causal attributions (biological/neurological or traumatic/lived experiences) for mental distress. This suggests a possible gap between the church communities aetiological understanding of mental distress and individual’s personal synthesis of aetiology and the resulting difficulties in assuming meaning. In their subsequent large-scale qualitative survey of 293 respondents, Lloyd and Hutchinson’s (in press) findings highlighted stigma and the totalising spiritualisation of mental distress as commonplace for British evangelicals with mental distress. Nevertheless, the authors reported that while the evangelical Christian faith could be experienced as both invalidating and dismissive for those living with mental distress, it could also function as a source of support.

### The Present Study

Together, these findings necessitate further research exploring individual meanings in regards to the interface between psychological suffering and Evangelical Christianity. As Lloyd and Waller (2020) suggest, there is a need for phenomenologically grounded qualitative research to inductively explore how evangelical Christians experience their mental distress within the context of their faith and wider communities.

## Methods

Christianity is the largest religion globally, with approximately 2.4 billion adherents, making up 31% of the global population (Pew Research Centre, 2015), with over 600 million identifying as evangelical. In the UK, 38% of the population identify as Christian, (Curtice et al., 2019), with an estimated 2 million identifying as evangelical (Evangelical Alliance, 2020). Despite their large presence in the UK and worldwide, we know very little about evangelical Christians’ lived experiences from their own perspectives.

### Research Design Overview

A qualitative framework was used as the basis for this research, which supported the acquisition of exploratory, non-directive research focused on experience and meaning-making rather than identifying cause-and-effect variables. Due to the limited availability of research exploring mental distress in evangelical communities, a qualitative approach was deemed a good fit for creating new knowledge (Smith et al., 2009). According to Willig and Rogers (2017), qualitative research is well-matched for an in-depth exploration, description, and interpretation of an individual’s experiences. For this study, IPA (Smith et al., 2009) was chosen as the most suited interpretive framework due to its in-depth exploration of how individuals understand their experiences (Pietkiewicz and Smith, 2014).

Smith (2004) demarcated IPA as embedded within three theoretical bases: phenomenology; hermeneutics; and idiography. IPA is based on the in-depth analysis of detailed personal accounts obtained from small homogenous samples. Unlike hypotheticodeductive methodologies (quantification and testing of variables to disprove scientific hypotheses), the aim is not to establish general laws, but instead to lay preference to an individual’s subjective experiences (Lyons and Coyle, 2016). Moreover, IPA’s hermeneutic position necessitates acknowledging the interpretative nature of research and use of the “double hermeneutic,” whereby participants draw on their own understandings to interpret their experiences, which through the researcher’s analytic process, becomes interrelated with their interpretative endeavours (Smith et al., 2009). While sample representation is not generally considered an issue in qualitative research, the utility of such small-scale qualitative studies is such that the knowledge and understanding they develop can later be subject to hypothesis generation and tested against the hypothetico-deductive paradigm (Willig and Rogers, 2017).

### Ontological and Epistemological Positioning

This study is positioned within critical-realism (Collier, 1994) as its ontology and phenomenology (Giorgi and Giorgi, 2003) as the epistemology. Specifically, through critical realism, this study assumes the existence of a material world outside of individual consciousness, yet which is only intelligible through examining individual accounts of those experiencing phenomena (e.g., mental distress in evangelical communities) (Giorgi, 2006). Thus, phenomenology provides the nearest epistemological fit, as it allows for the exploration of accounts from those experiencing the phenomena in their own words and on their own terms (Pietersma, 2000).

### Reflexive Statement

A major principle of qualitative research is the significance assumed between researcher subjectivity and research (Willig and Rogers, 2017). As such, no researcher can claim impartiality or objectivity of truth. Instead, researchers are positioned as co-constructors of knowledge (Gergen, 1985). In an attempt to restrict the influence of such processes, transparency of approach is advocated for. This has been termed reflexivity, characterising the researcher’s process of acknowledging their presuppositions and personal interests as they relate to and arise within the generation of new knowledge (Berger, 2015). The author was raised in an evangelical Christian home, which emphasised the Holy Spirit’s importance in all aspects of Christian life. This early exposure to theological and spiritual understandings of complex problems in living has influenced the author’s appreciation for the role of the spiritual in mental distress. To restrict the influence of such lived experience on the present research, a reflective diary was maintained throughout data collection and analysis to bracket presuppositions with regards to how participants were experiencing phenomena.

### Participant Recruitment

This study was aligned with the British Psychological Society's Code of Human Research Ethics (2014) and received full ethical clearance from the University of Oxford prior to study commencement. Informed consent was obtained from each of the study’s participants. Participants were recruited online from a large-scale mixed-methods survey that explored mental distress in evangelical communities (Lloyd and Waller, 2020). This survey was advertised online through various evangelical social media groups that offered to share the survey link with their subscribers and with subsequent snowball sampling. The survey was advertised as a “short online survey that aims to capture your experiences of churches’ attitudes toward your own and others’ mental health issues” (see Lloyd and Waller, 2020, p.4 for further details). Upon completion of the survey, participants were invited to leave their contact details if they were interested in engaging in a subsequent exploratory one-to-one interview concerning their experiences. Twelve participants left their contact details, of which eight consented to a follow-up interview.

### Study Sample

This study was conducted in the United Kingdom between 2018 and 2020, consisting of eight self-identified evangelical Christians (Table 1), aged 35 to 64 (M = 49.5 years, SD = 10.6 years). Four participants identified as male, while four identified as female. All participants were Caucasian and resided in the United Kingdom at the time of participation. Four of the participants attended church/religious services weekly; one did so more than five times weekly; one twice weekly; one monthly, and one was not attending at the time of interview. To provide satisfactory homogeneity as required by IPA (Smith et al., 2009), all participants self-identified as evangelical Christian (M = length of faith adherence, 32.9 years). All identified as previously having had a formal mental health diagnosis, ranging from anxiety and depression to personality disorder, which was provided by either primary or secondary care psychiatric services. All participants had broadly sought help from the church in relation to their mental distress. These help-seeking pathways were broadly defined and included, informal church interaction, such as social conversations in church services with other members and leaders, as well as, receiving spontaneous prayer during services. More formal interaction included individual meetings with Christian faith leaders to formally discuss and seek support for their mental health.

**Table 1:** Participants’ demographic information (pseudonyms used to maintain anonymity)

Pseudonym	Mick	Dorothy	Simon	Timo	Shan	Angela	Victoria	Raffaello
Age	63	36	64	50	41	35	55	52
Gender	Male	Female	Male	Male	Female	Female	Female	Male
Length of Adherence to Faith	46 years	15 years	40 years	45 years	30 years	10 years	38 years	39 years
Route to Help-Seeking	Informal church interaction	Informal church interaction	Informal church interaction	Informal church interaction	Informal church interaction	Informal church interaction and 1–1 church ministry	Informal church interaction	Informal church interaction
Mental Health Diagnosis	Depression and anxiety	Anxiety and panic attacks	Anxiety and depression	Post-traumatic stress disorder, anxiety and depression	Post-natal depression, anxiety and depression	Borderline personality disorder	Complex post-traumatic stress disorder	Depression
Medication	Yes	No	No	No	No	Yes	Yes	No
Frequency of Church Attendance	5 + weekly	Weekly	2 + weekly	Weekly	Weekly	Weekly	Monthly	Not currently attending

### Data Sources and Collection

In aiming to inductively explore how evangelical Christians experienced and made sense of their mental distress in relation to their faith communities, it was anticipated that the use of semi-structured interviews with open-ended questions and probes would permit for a rich and contextualised dataset (Smith et al., 2009). All interviews were conducted online via Skype at a time convenient for participants and were in their own residences when participating in interviews. Interviews varied between 30 min and one hour in length (M = 36.6 min). As a psychologist, the author has advanced training and experience working with and containing psychological distress and ensured that all participants were comfortable responding to questions throughout the interview process. The interview schedule (Table 2) included open-ended questions drawn from the available literature. Consistent with qualitative research principles, the schedule was used to guide the researcher-participant dialogue rather than for formulaic use (Willig and Rogers, 2017). Interviews were audio-recorded and transcribed verbatim.

**Table 2:** Semi-structured qualitative interview schedule with prompts

1. As a Christian, what do you understand as the possible cause(s) of mental illness? *Has this always been the case?* 2. How do you feel the church generally conceptualises mental health? *Could you give me some examples?* *What has been the effect of this for you?* 3. Can you tell me about your experiences of mental distress and Christianity? *Could you tell me more?* 4. Can you tell me about your experiences of mental distress in relation to your current or previous congregation? *Any positive or negative experiences?* *What has been the impact of these experiences?* 5. How do you feel others in the church have interacted with you? *In relation to your mental health?* *Could you give me some examples?* *What has been the impact of these experiences?* 6. How and in what ways do you feel the bible conceptualises/talks about mental health? *Is this any different or similar to the church?* *Or you own understanding and experience?* 7. What in your mind would be the ideal Christian response to individuals with mental health conditions? 8. Do you have anything to add, or to share, that you feel is important or that you think this research should hear?	

### Data Analysis

The analytic procedure was guided by Smith et al.’s framework (2009). Repeated reading and re-reading of each individual transcript was completed with extensive notes and connections made in each transcript. These notes captured descriptive (the content of participants’ speech), linguistic (specific language used, such as metaphors and notes on conceivable function), and conceptual meanings (additional interrogative depth used to comment on possible underlying meanings). This involvement in the data resembled a form of Gadamerian dialogue; that is, the interrelation of the researcher’s pre-understandings and newly formed understandings from immersion in the data (Smith et al., 2009). Meanwhile, abstract notes and psychological concepts were noted, functioning as an analytic shift from working with the transcript to working with emerging themes. Theme clustering involved seeking areas of convergence and divergence across emerging themes in order to establish a coherent story concerning participants’ experiences. This culminated in the production of a master table (Table 3) with superordinate and subordinate themes and exemplar quotes which aimed to capture the essence of each theme.

### Assessing Validity and Quality

Yardley’s (2008) quality criteria for qualitative research were employed to ensure research quality. Firstly, “sensitivity to context” incorporated sensitivity to and awareness of the existing research literature, including ensuring that all data analysis was grounded in each participant’s feedback and answers. This process was also supported with participant theme validation, whereby participants were individually approached to review the emerging themes and offer feedback on their validity and resonance (Sousa, 2014). All participants were approached following initial data analysis, with two out of eight participants responding to the request for feedback on the themes. Both of the participants who responded to the request for theme validation were satisfied that the emerging themes authentically captured their experiences and suggested no changes.

Secondly, “commitment and rigour” was achieved by attending carefully to participants’ dialogue during interviews, allowing for their experiences and phenomenological accounts to emerge inductively. In accord with Yin’s (1989) proposal, a “paper trail” was amassed during the analysis process to support on-going reflection. This included utilising a reflective journal to bracket the researcher’s assumptions and prevent undue interference in the interpretative stage. To improve the study’s “transparency and coherence,” step-by-step procedures were utilised and are provided in detail in the methods section, alongside a brief reflective account from the author. Finally, criteria for “impact and importance” were met by the researcher’s commitment to addressing an important gap in the literature, and by carefully considering and drawing out the potential consequences for individual and collective psychological and spiritual well-being.

## Findings

Two superordinate themes and seven sub-themes emerged from the detailed idiographic case-by-case analysis of each narrative interview (Table 3). In accord with IPA principles, each superordinate theme is discussed below, with sub-themes and exemplar quotes demonstrating how participants made sense of and experienced their mental distress in relation to their evangelical Christian faith and community. Pseudonyms have been used throughout to preserve participant anonymity.

**Table 3:** Superordinate themes and subthemes, with exemplar abridged quotes

Superordinate themes	Subthemes	Key quotes
Negative spiritualisation	Equating mental distress as symptomatic of the demonic	“… I was even once described as demonic…they decided that I was demonic and I was demon possessed, which is why they then went ahead with this deliverance…”. Angela
	No language for mental distress	“… There was no language, there was no language to talk about it in relation to faith…”. Mick
	Dismissing secular intervention	“Sometimes the attitude will be you don't trust secular psychology… It's doesn't have a Christian basis. Maybe for that reason”. Simon
	Questioning the (non) miraculous	“They were very, very big into signs of wonders, healing, and that kind of thing. If you were feeling that you had bad mental health and you went forward at a Wimber meeting and you weren't healed, you felt pretty shit really…”. Mick
	A climate of stigma and shame	“I've found it very difficult to be at church. Sometimes it's easier to get there late and then leave early or leave as soon as it finishes, just not to talk to people…because I think that they'll judge me”. Timo
Negotiating the dialectic between faith and lived experience of mental distress	Rejecting an either/or aetiological understanding of mental distress	“I don't think that's the case. I think we're very complex. We're body, soul, and spirit. Any one of those three areas could cause problems…”. Simon
	The case for holistic and inclusive treatment	“…I really think that the physical and spiritual is really interconnected, and so I would want to be talking to somebody who has that understanding…” Dorothy

### Negative Spiritualisation

Eight participants referred to situations when they had directly experienced negative interactions in relation to their mental distress from within their Evangelical Christian communities. These negative experiences connected with what seemed to be experienced as spiritually reductive experiences (all phenomena and experiences as attributable solely to spiritual agents), through which the wider church made sense of each participant’s mental distress.

### Equating Mental Distress as Symptomatic of the Demonic

All eight participants expressed concern for how their community of faith made sense of mental illness. In particular, participants referenced comments or remarks made by their church leaders or ministers equating their mental distress as a direct consequence of demonic infiltration. This seemed to be experienced as unhelpful, dehumanising, and frequently superimposed, feeding into the very distress participants sought support for. Angela remarked:

Of them saying that I'm not mentally ill, I'm possessed, that it's an evil spirit... I was even once described as demonic… they decided that I was demonic and I was demon possessed, which is why they then went ahead with this deliverance. That just distressed me more and really did distress me. (Angela)

Here, Angela describes the response she received from her church leaders in relation to her mental health when she went forward for counselling from her church. From her remarks, it seems her church community responded to her mental distress with an uninvited spiritualisation, whereby an “evil spirit” or external agent was located as the cause of her suffering. Her use of “they then went ahead…” seems to underline the potentially forced nature of this intervention, as if Angela had not welcomed this worldview or intercession. Furthermore, her language points to the presence of power and control, where church community disregards her autonomy by going “ahead” with the deliverance instead of being asked to intervene.

Perhaps assumptions of demonic interference from the church functioned to justify their imposed interventions with Angela. She further remarked, “that just distressed me more…” indicating a sense of helplessness that forced spiritualisation can bring for those struggling with their mental health. Angela’s experiences were shared with other participants too; Victoria, for example, shared the following:

I went forward and asked for prayer and, in fact, the person who came to speak to me was the head of the pastoral team and used to be a church minister. I said that I was really very low and very depressed or feeling suicidal, and he just literally stood over me and kind of [denounced] the devil, and sort of praying the devil leaves me… it made me feel awful, absolutely awful. (Victoria)

…because I disagreed with certain things that they were saying, [the minister] actually told me, I let the enemy into my head, I was listening to the enemy. I wasn't allowed to talk to anyone, I was quite isolated, very manipulative and controlling behaviour. (Victoria)

Victoria describes a previous church minister’s response to her seeking support for her low mood and suicidal thoughts. Similar to Angela’s experience, Victoria’s minister seemingly locates her distress as resulting from internal demonic activity. This is evidenced by their attempts to expel the demon by “praying [that] the devil leaves me.” As Victoria explicitly remarks, equating her psychological distress exclusively in spiritual terms left her feeling “awful.” Perhaps her use of “awful” points to her discomfort and the potentially negative effects, a totalising spiritualised aetiology for mental distress can bring.

#### No Language for Mental Distress

In describing church teachings and interactions, which reduced participant experiences of mental distress solely to the demonic, four participants described a wider cultural milieu, which lacked an understanding of mental health beyond spiritualised onto-aetiologies. Mick felt that the problem was a lack of nomenclature in his church when it came to discussing mental health concerns. He shared,

I think there were very few people in those churches who understood [mental distress]. If they did know something about it, they didn't have any way of [expressing it]. There was no language to talk about it in relation to faith. There was no framework for those conversations to take place. (Mick)

Mick highlights the lack of “framework” for discussing mental health in his church, which seems to preclude conversations about mental health taking place, as members of his church have “no language to talk about it in relation to faith.” As Mick elaborates, this lack of language for making sense of mental health and distress seems to result in a theology that assumes spiritual causality. He notes,

The go-to explanation for anything that's unpleasant or unwanted or difficult is to regard it as a demonic attack. Those churches, if not wisely guided, do tend to have a rather positional [attitude]; “this is God and all the good, and there's the devil and all the bad, and mental health is bad, so that must come from the devil.” It's easy, but it can lead to a lot of very damaging behaviour. It could lead to people thinking what they need to do with people who are mentally ill is exorcise them. (Mick)

#### Dismissing Secular Intervention

The dismissal of help-seeking outside communities of faith was another difficulty experienced by three participants and led to negative experiences concerning mental health. As Simon remarked, “sometimes the attitude will be you don't trust secular psychology… They’d probably say it's because it's based on materialism or something. It's doesn't have a Christian basis.” Simon highlights what he believes to be the broader evangelical church’s suspicion of secular or professional help-seeking, which he attributes to the perceived lack of a Christian basis present in psychology and secular systems of support. Meanwhile, Angela noted an impetus towards rejecting biomedical interventions and relying solely on spiritual interventions, such as prayer: “To be honest, it wasn't very helpful, because they believed that I didn't need any medical help. I didn't need to be on medication, that it could all be dealt with prayer and various other things.” (Angela).

#### Questioning the (Non) Miraculous

Eight participants spoke of how in addition to the assumption of a spiritualised aetiology for their mental distress, an imperative or expectation of healing emerged. Prayer and healing were demarcated as the only solution to psychological suffering:

They were very, very big into signs of wonders, healing, and that kind of thing. If you were feeling that you had bad mental health and you went forward at a Wimber meeting^3^ and you weren't healed, you felt pretty shit really. It did not really help your poor mental health. (Mick)

If only you prayed more, if you only read the Bible more, surely, this wouldn't be happening. It happens anyway*.* (Dorothy)

Mick and Dorothy seem to highlight the miraculous expectation through their narratives, which was also presented in other participants’ experiences. For eight participants, a cultural expectation of healing for mental illness existed in their church communities, such that if an individual failed to receive divine healing for their suffering, their faith status and quality of spiritual life would be questioned. It seemed there was no space for a theology of suffering in these instances. As Mick seems to highlight, the cumulative effect of such expectations for healing was often one of disappointment: “If you went forward at a meeting and weren’t healed, you felt pretty shit really.” In these instances, blame for not healing was often shifted towards the individual suffering from distress or was internalised by the individual due to questioning from their wider faith community. Dorothy states, “a question that not everyone but some people have asked is, ‘How's your walk with Jesus going?’ They think that it correlates.”

#### A Climate of Stigma and Shame

Two themes common throughout participant responses were references to direct and implicit stigma and shame with respect to experiences of mental distress. For all participants, the cumulative effects of having their mental distress associated solely with demonic origins, the lack of a framework or language for discussing mental distress and suffering, and the dismissal of secular interventions and push for miraculous healing, seemed to lend itself to a climate of stigma and shame. Ultimately, this exacerbated the psychological difficulties for participants.

Simon describes the lack of dialogue in his church and the ‘sidelining’ of people with mental health problems. For people struggling with mental distress, this seems to result in social rejection. As Simon pointed out, “There was no serious dialogue in the evangelical churches I was part of about mental health. People who had mental health problems were sidelined, I think, is perhaps the best way to put it.” Meanwhile, Mick discusses not receiving the healing that he expected from his faith community and their theology. This seems to have resulted in a climate of shame, whereby it became difficult to discuss or share personal struggles with others, leading to relational disconnection. Climates of shame appeared to be the case when dichotomies were assumed between good and evil or psychological distress and well-being. Conducive to Mick’s experience, theological significance was given exclusively to health and happiness:

I felt very distressed by that period of ill-health. I was in my early-mid 30s and felt that I'd kind of given everything to God, and I'd been let down. It was very difficult I didn't really feel I could share how I felt with any evangelical church. I had to go and play along. There's quite a lot of playing along in those communities I found to fit into their requirements about being people of faith, and confidence and trusting God and all that kind of thing*.* (Mick)

Timo also shared his experiences where he felt uncomfortable in his church community, fearing judgement from others about his mental distress. For Timo, social isolation was an effect of such spiritualised worldviews. He stated:

I've found it very difficult to be at church. Sometimes it's easier to get there late and then leave early or leave as soon as it finishes, just not to talk to people… because I think that they'll judge me*.* (Timo)

### Negotiating the Dialectic Between Faith and Lived Experience of Mental Distress

For every participant, the negative and reductive spiritualisation they experienced in their church was a point of tension. Specifically, participants rejected what they experienced as dominant, deterministic spiritualised aetiologies of mental distress. Instead, they sought to develop and negotiate their own theological synthesis of psychological suffering, incorporating and honouring the reality of their own lived experience alongside dedication and commitment to their Christian faith.

#### Rejecting an Either/Or Aetiological Understanding of Mental Distress

By making sense of their own experiences and understandings of mental distress, eight of the participants rejected a solely spiritualised aetiological understanding of psychological suffering. In its place, participant accounts appeared to emphasise a more multifaceted understanding of mental distress, which also accepted some degree of uncertainty in knowing or determining exact causes.

I don't think that's the case [that the only cause of mental distress is spiritual]. I think we're very complex. We're body, soul, and spirit. Any one of those three areas could cause problems. It might be a physical thing, or it might just be psychological or it might be spiritual, and it's sometimes difficult to know which one is the case. (Simon)

In attempting to make sense of human nature and psychological distress, participants appeared to rebuff theological teachings or practice, which prioritised spiritual understandings, with neglect to their physical, psychological and social intersections. These accounts typically represented a departure from exclusively spiritual onto-aetiologies, towards acknowledging a variety of factors, causes, and contexts.

I feel like, when I read the Bible, it's very holistic… I think that God made us, and that He made us knowing that we're physical. [T]here's chemicals in our bodies and all sorts of stuff behind it [speaking of causes of mental distress], I’m personally okay with knowing that trying to understand mental health from a Biblical perspective has to be more than just spiritual. (Dorothy)

While all eight participants were keen to reject rigid spiritual accounts of mental distress, this often did not represent a complete move away from emphasising the possible impact of the spiritual realm on well-being but rather embraced understandings and awareness of how all parts of a person were connected with one another, including the spiritual, as Shan shared:

I think the whole cause of mental health is complex. I think it's probably a mixture of all of those things [physical, psychological, social and spiritual]. My personal view is the enemy uses our weaknesses and tries to tempt us… Yes, that's part of living in a fallen world*.* (Shan)

#### The Case for Holistic and Inclusive Treatment

Participants generally moved to integrationist aetiological understandings of mental wellness and suffering. That is to say, participants valued a response that recognised interconnection and a variety of factors influencing psychological well-being. Four participants voiced their views on suitable treatments and interventions for mental health concerns:

I believe in medication [laughs]. I believe that it's incredibly beneficial and more than that, I think in some situations it’s absolutely necessary… I believe in praying, I believe in the spirituality behind it, I believe that Jesus can heal, I really do, but I also believe in the same way I would that if somebody came and told me that they've been diagnosed with cancer, I would say, “We're going to pray for you. I'm going to pray and ask God to give me faith to pray for your healing, but please go see a doctor, get all the treatment you physically need.” I'm of the understanding that, “Yes, let's pray,” but the spirit and physical are interconnected… and there are doctors around us, and thank goodness for doctors, and God has blessed people in that area. (Dorothy)

For all eight participants, recognising the value of secular help-seeking, such as visiting a doctor, therapy, and medication, were positioned as equally valuable treatment options in addendum to prayer. As Dorothy remarked, secular help-seeking and prayer were understood as necessary for aiding mental well-being, rather than seeking one or the other in isolation. For Raffaello too, it appeared that negotiating his experience with mental distress and theology led to an understanding of the need for inclusive support, which moved beyond mere dichotomies of spiritual versus secular:

I actually came to the conclusion that most mental health issues have got the root of the attachment issues. That actually good healthy attachments with God and with people will keep us from [distress]. If we have those all the way through our lives, we wouldn't have mental issues in the first place. That is also the vehicle of healing. You need good healthy attachments to go through stages of development and sometimes what's broken in us can be healed through a good relationship with God and people. (Raffaello)

[I]t would be to recognise that the medical and the psychological help can work hand in hand with Christianity and prayer in faith. (Angela)

Both Raffaello and Angela appear to emphasise the value of relationships, with both “God and people,” for healing. Raffaello seems to identify disrupted relationships as a possible cause of future mental distress. Indeed, his recognising the value of “healthy attachments” indicates the need to include both spiritual intervention and relational support. Angela, too, emphasises the significance of departing from reductionist or isolated spiritual or secular paradigms towards a paradigm that integrates spiritual help and social connection. Participants emphasised that such perspectives could offer significant and positive value to evangelical Christians struggling with mental distress.

## Discussion

This paper has drawn on phenomenological methods to explore how eight evangelical Christians with lived experiences of mental distress in the UK made sense of their suffering in relation to their faith and church communities. This was thought to be particularly salient considering the growing body of literature highlighting the dangers of excessive spiritualisation and stigmatisation towards those with mental distress in evangelical Christian communities (Leavey, 2010; Lloyd and Waller, 2020; Lloyd and Hutchinson, in press; Scrutton, 2020; Weaver, 2014; Webb et al., 2008; Webb, 2017; Wesselmann and Graziano, 2010). While the majority of previous research has drawn almost exclusively from an American context (Stanford, 2007; Webb, 2008, 2017), the present study has explored mental distress within a British Evangelical context. Following the survey findings from Lloyd and Waller (2020) who first explored aetiological understandings of mental distress in a sample of British evangelicals, and more recently, Lloyd and Hutchinson (in press) who utilised a qualitative survey design and thematic analysis to identify both positive and negative aspects of spiritualisation in evangelical communities, this phenomenological study sought to further explore these themes. While the findings from the present study are qualitative and do not attempt to delineate causal pathways, nor do they allow generalisation to other Christian communities, they do afford a helpful preliminary understanding of some of the pertinent issues and psychological processes evangelical Christians with lived experience of mental distress may be exposed to.

### Contending with a Reductive Spirituality

Firstly, results from this study indicate that spiritual onto-aetiologies concerning mental distress remain for some evangelical communities, even within a UK context.^4^ This is broadly consistent with prior psychological research (Leavey, 2010; Lloyd and Waller, 2020; Lloyd and Hutchinson, in press; Meyer, 2001; Weaver, 2014; Webb et al., 2008; Webb, 2017). The present study’s results help to further extend these findings by illuminating how the spiritualisation of mental distress becomes unhelpful in church communities.

The qualitative interpretation of the participants’ interview data seemed to suggest their church communities often responded to their experiences of mental distress by equating their suffering as connected primarily with demonic or spiritual involvement (spiritual reductionism), alongside the wider cultural expectation of healing. This entailed experiences where the church often inappropriately used prayer to expel demons or to insist on prayer alone as the route to healing. This uninvited use of deliverance also seemed deeply connected to expectations of healing from the broader church milieu. In other words, those in the church community who prayed and anticipated immediate solutions for psychological suffering would often look for evidence of healing or change in the individual. From a psychological perspective, these processes may well indicate confirmation bias, whereby individuals search for, interpret, favour, and recall perceptions or information in a way that confirms or supports existing prior beliefs or values, while minimising evidence which contradicts dominant beliefs (Nickerson, 1998).

If healing or deliverance from mental distress was not evidenced, participants described exposure to implicit attitudes, which questioned the reasons for the lack of healing. This very often involved some absorption of internalised guilt and shame by the individual, or at the very least, a deeper questioning of their personal faith. As Dorothy remarked, “A question that not everyone but some people have asked is, ‘How's your walk with Jesus going?’ They think that it correlates.” For participants, such questioning often attacked the core of their faith, by attempting to imply that the presence of mental distress was indicative of reduced or weakened faith on their part.

From a cognitive-behavioural perspective (Beck and Beck, 2011), demonic accounts of mental illness are also likely to be unhelpful if they reinforce negative internal beliefs that individuals hold about themselves. Thus, it seems that through this lens, demonic accounts may be pathogenic in and of themselves if, as was the case for several participants in this study, individuals are labelled demonic due to their struggles with their mental health or exhibit behaviours attributed to demonic influence (Scrutton, 2020).

The experiences of participants in this study seem in many ways closely aligned to the work of liberation and disability theologies, which offer a caution against assuming that the healing ministry of Jesus provides an exact template for the Church’s response to people living with mental distress, as doing so excludes those who do not receive healing (Cook and Hamley, 2020). Disability theologian Eiesland (1994) argues that insisting upon healing as spiritual normalisation and confirmation of faithfulness is deeply oppressive for those desiring both theological and human authenticity in their lives amidst personal psychological suffering. Indeed, this finding is largely concurrent with Lloyd and Waller (2020), who reported that 31% of evangelical Christians had experienced church teachings that positioned deliverance and prayer as the *sole* cure for psychological suffering, along with Lloyd and Hutchinson (in press), who identified imposed spiritualisation as a general theme emerging from 293 participant responses.

Furthermore, spiritualised hermeneutics for mental health provided by church communities, often carry an idiosyncratic meaning for participants. Specifically, Lloyd and Hutchinson (in press) discuss how the spiritualisation of mental distress can function to alleviate but also further suffering. Within the current study, it appears that for all, the consequences of being labelled “possessed” often furthered psychological distress for individuals when they sought support. These experiences are also concordant with the theoretical concepts of positive and negative religious coping (Pargament, 1997). Furthermore, evidence of the perils of a stigmatised context is comparable with the literature, which suggests that religious beliefs are positively related to stigmatising beliefs regarding mental illness (Wesselmann and Graziano, 2010). In other words, advocating for certain beliefs (e.g., that mental illness/distress results exclusively from immorality/sinfulness, or that mental illnesses have spiritual causes and treatments) have been found to predict a preference for giving spiritual social support (Rogers et al., 2012; Stanford, 2007; Stanford and McAlister, 2008). All participants noted that proposed spiritual aetiologies were often presented in dichotomised terms (e.g., good versus evil, demonic versus Christian). Both Mick and Shan experienced this when they referenced being exposed to expectations of healing from mental distress (health versus ill-health). Shan expressed her desire to move away from the binary of spiritual versus secular care to a place where both approaches authentically captured her experiences while remaining true to her Christian faith. From a psychological perspective, binary modes of thinking often emphasise extremes, superimpose a value hierarchy, negate nuances of meaning, and close-down possibilities for understanding and action (Berlin, 1990). By contrast, more nuanced explanations often capture context and hence represent more holistic accounts of phenomena.

Furthermore, in this study, the risk of experiencing negative emotions seemed to be heightened when participants’ lived experience or aetiological understandings were dismissed or stigmatised by members of their church community, which in turn restricted their own sense-making and “othered” them as human beings. This perhaps points to the process of “othering,” which was initially recognised within disability literature to refer to how individuals with illness may be objectified into an object of difference, such as a stereotype (Richards, 2008). In the present study, Timo reported this vividly when he described experiencing relational disconnection from his church community due to real or perceived fears of judgement, which ultimately perpetuated his suffering by maintaining negative core beliefs about himself. Indeed, a useful theoretical framework for interpreting the effects of a spiritualised understanding of distress, which participants were subject to, parallels what is termed a “dividualising” experience, whereby an individual’s identity is subject to fragmentation (Colwell, 1996). Allen and Brown (2016) argue that a dividualising process results in the implementation of strategies that dissolve the specificity of individual life and experiences. Thus, as this study demonstrates, rather than supporting individuals to make sense of and recover from their distress in their own terms, the church community directs their care towards spiritual aspects and disregards the whole context of an individual’s distress. This seemed to be experienced by Angela, Dorothy, and Victoria when they laid bare their distress only to have it interpreted as the result of demonic activity. Other sources of care and nurture were further discounted. For this study’s participants, this seemed to culminate in a fragmenting or dividualising of the person along with their personal, psychological, and social history being split from their faith and spiritual lives.

### Negotiating the Dialectic Between Faith and Lived Experience of Mental Distress

In making sense of their own experiences and understandings of mental distress, all the participants rejected a solely spiritualised aetiological understanding of psychological suffering. Instead, participant accounts emphasised a more contextualised and holistic position, which seemed to be undergirded by participants’ own attempts to synthesise secularised understandings with their own theological authenticity (Leavey, 2010), demonstrating an integration of biopsychosocial, religious, spiritual and cultural discourses. These accounts often represented a marked shift from dominant evangelical church teachings and praxis, with associated totalising claims to truth (e.g., all mental distress is demonic), viewing suffering in more contextual and integrationist terms (i.e., biopsychosocial alongside spiritual). Perhaps, the adoption of an increasingly contextualised aetiological understanding of mental distress within evangelical communities, parallels developments in wider evangelical theological praxis, such as the growing acknowledgement and criticism of the “prosperity gospel” (Jones and Woodbridge, 2011). The prosperity gospel is defined as a religious belief system specifically prominent among evangelicals, which argues that well-being and economic success are unilaterally the will of God and that faith, self-affirmation, and financial endowments to religious communities will manifest individual health and wealth. Recently, such teachings have been critiqued by theological scholars (Fortner, 2016) owing to its denial of the present and unavoidable reality of suffering (including psychological) all humans face.

## Conclusion

While perhaps, none of these results are necessarily unexpected in view of how Evangelical Christian healing practices tend to emphasise the role of supernatural agents (God versus the demonic) in causing suffering (Leavey, 2010); the present study does highlight some important psychological processes worth consideration. Specifically, this paper evidences that the spiritualisation of mental distress may be more likely to be received or understood negatively when individuals have not embraced a spiritual frame of reference themselves (e.g., when this is imposed or issues of control and power are present) (Oakley and Kinmond, 2013). Moreover, the negative impacts appear heightened when the spiritualisation of suffering contributes to the creation of a stigmatised identity, which leads to shame and relational disconnection from others (Lloyd and Hutchinson, in press). The process of othering likely furthers psychological suffering by removing important sources of social capital and meaning, which are vital to well-being (Rogers, et al., 2012; Stanford, 2007). It is possible that this carries a self-fulfilling effect. Indeed, a particularly noteworthy finding in the present study was how participants’ disagreement with or withdrawal from church life was interpreted by the wider church community. It seemed that this was taken as further evidence of demonic involvement. Victoria, for example, experienced this directly, when church leaders equated her disagreement of their views with letting “the enemy into my [Victoria’s] head.” This further signals the need for increased mental health literacy in evangelical Christian communities in order to counter mental health stigma and oppressive forms of doctrine, which promote individualised and interiorised simulacrums of the gospel. Such harmful perceptions can be replaced with spiritually syntonic forms of relational support and care.

As the present study focused on providing a detailed and localised subjective interpretation of a small number of participants, generalised conclusions from this study to all evangelical populations are limited. Likewise, this study does not claim that the experiences of the present sample can be extrapolated to all evangelicals.^5^ Nevertheless, based upon the participant experiences, phenomenological analysis and literature, the following tentative conclusions and contributions to knowledge are made regarding the present study sample, which require further empirical investigation:

1. Evangelical Christians with mental distress may be at increased risk of encountering theology, church teaching, or ministry, which locates their mental distress as principally spiritual in origin (e.g., demons or spirits, generational curses, personal sin).
2. The individual meaning of this spiritualisation is deeply idiosyncratic and assumes a localised moral, personal and theological significance but may increasingly carry a negative implication for individuals, if this is imposed onto the individual and/or is not in line with the individual’s own sense making.
3. If the spiritualisation of mental distress does occur, it may also lead to the creation of a stigmatised identity and context (e.g., “you’re demonic”), which increases self-stigma, prevents relational connection, and leads to the maintenance and/or generation of further psychological distress.
4. Evangelical Christians with mental distress may seek to make sense of their psychological distress through both naturalistic (biological, psychological, social) discourses, as well, as spiritual and theological narratives, with many rejecting and departing from dichotomised aetiological understandings of psychological distress (e.g., as either psychological or spiritual).

### Reflections on Study Design, Limitations and Further Research

In order to support the development of further research, there are a number of strengths and limitations arising from this study that merit discussion. Firstly, this study’s strength relates to its use of the phenomenological method, which prioritises attempts to understand participants’ experiences inductively, on their own terms rather than imposing or assuming meanings (Smith et al., 2009). The aims of such an approach also usefully allows for the later employment of hypotheticodeductive methods, which are capable of building upon qualitative findings through nomothetic variables measurement, or the delineation of causal relationships. Further research might include exploring the effects of stigma and shame on psychological functioning and recovery, or what might predict solely spiritual aetiological understandings of mental distress.

Additionally, as this study built on earlier research (Lloyd and Hutchinson, in press) by showing how and in what forms reductive spiritualisation becomes unhelpful for individual and collective well-being in evangelical Christian communities, further research may seek to create valid and reliable psychometric assessment tools for use in such contexts. Such tools could usefully delineate who is at risk of stigmatisation or where internalised aetiological beliefs regarding mental distress might create further challenges to well-being. It would also be noteworthy to explore the individual negotiation that takes place following experience of negative spiritualisation, such as potential reasons for individuals to remain, or to leave, their evangelical religious context. Both qualitative and quantitative designs would be helpful in this aspect.

Furthermore, due to some potential power issues that emerged in this study, subsequent research might explore whether demonic interpretations of mental distress are connected to (a) perceptions of the church’s responsibility/obligation to act or (b) to an individual’s perceived ability to choose a particular intervention. Although the present study lacked the necessary research design to determine whether there was a gendered aspect to having a spiritual aetiology of mental distress imposed, Scrutton (2020) has gestured towards a correlation between the attribution of sin/demonic aetiologies and intersectional variables, such as sex, gender or sexuality. Flowing from this, it would be useful for further research to explore whether intersectional aspects of identity, such as gender or sexuality, may be associated with increased likelihood of having a spiritual explanation for mental distress imposed by the church.

Additionally, while this research has shown that the spiritualisation of mental distress can be problematic under certain conditions, it would also be useful to explore how and in what forms demonic, sin or other spiritual accounts of mental distress may be helpful or adaptive for individuals and communities (Nelson and Koetke, 2018). This should include actively recruiting participants who subscribe to demonic aetiologies, as well as, those who are critical. Further qualitative and quantitative research could fruitfully explore this. Interdisciplinary input from theology and philosophy would also be useful in this respect to critically evaluate the forms of negative spiritualisation discussed in this study.

Finally, it is well acknowledged that evangelical communities represent a heterogeneous group, encompassing a variety of beliefs and practices (Hackett and Lindsay, 2008; Lancaster et al., 2019). This has implications for research and can problematise the operationalisation of terms such as “evangelicalism” or “evangelical,” often adding obstructive heterogeneity into study samples. Although all participants in the present study self-identified as evangelical, to satisfy research study requirements for homogeneity of the sample, subsequent studies should utilise participant endorsement of creedal statements of faith as a condition of participation (see Bebbington, 2003; Evangelical Alliance, 2020).

## Data Availability

Due to the highly sensitive and idiographic nature of this study, interview data will not be made available.

## Footnotes

1 From the fundamentalist Christian perspective, the root of psychology is seen as polluted due to its adoption of naturalistic discourses and foundations in Pagan thought (e.g., Socrates, Aristotle, and Plato) (Yarhouse et al., 2016). Meanwhile, Freud’s view of religion further frustrated the appreciation of religious thought and practice. Specifically, Freud (1927, 2012) initially interpreted religion as cultural neurosis and Oedipal fixation on a powerful father figure.

2 Evangelical Christianity is operationalised at the trans-denominational level of belief system and praxis, with denominational affiliations such as Pentecostal subsumed under this (e.g., evangelical identification, with added emphasis to the power and role of the Holy Spirit).

3 A Wimber meeting refers to a charismatic evangelical gathering which stressed the role of the Holy Spirit, prayer and praise and the expectation of God’s healing. Wimber meetings were pioneered by John Wimber in the United States, in the 1970′s, with many attracting large numbers of Christians. For a critical discussion, see Benn & Burkill (1987).

4 It should be noted that there is generally a distinction drawn between British Evangelicalism, which is considered largely more liberal than its American counterpart, which is historically more fundamentalist (Marsden, 1977).

5 It is worth noting that the evangelical tradition is heterogenous in nature and hence there are likely to be a variety of conceptualisations of mental distress, which coexist, and extend beyond solely spiritual aetiologies. For further reading see: Cook & Hamley (2020); Scrutton (2020); Swinton (2017); Webb (2017).
